# Mobile App Support for Cardiopulmonary Resuscitation: Development and Usability Study

**DOI:** 10.2196/16114

**Published:** 2021-01-05

**Authors:** Sune Dueholm Müller, Kasper Glerup Lauridsen, Amra Hadrovic Palic, Lotte Nygaard Frederiksen, Morten Mathiasen, Bo Løfgren

**Affiliations:** 1 Department of Management Aarhus University Aarhus Denmark; 2 Department of Medicine Randers Regional Hospital Randers Denmark; 3 Research Center for Emergency Medicine Aarhus University Hospital Aarhus Denmark; 4 Department of Anesthesiology and Critical Care Medicine Children's Hospital of Philadelphia Philadelphia, PA United States; 5 Research and innovation Business Academy Aarhus Viby Denmark; 6 Department of Clinical Medicine Aarhus University Aarhus Denmark

**Keywords:** the Kano model, cardiopulmonary resuscitation, healthcare, smartphone apps, public health, ALS CPR algorithm, app evaluation, mobile phone

## Abstract

**Background:**

The user requirements for in-hospital cardiopulmonary resuscitation (CPR) support apps are understudied. To study usability, functionality, and design based on user requirements, we applied a mixed methods research design using interviews, observations, and a Kano questionnaire to survey perspectives of both physicians and nurses.

**Objective:**

This study aims to identify what an in-hospital CPR support app should include to meet the requirements and expectations of health care professionals by evaluating the *CprPrototype* app.

**Methods:**

We used a mixed methods research design. The qualitative methods consisted of semistructured interviews and observations from an advanced life support (ALS) course; both provided input to the subsequent questionnaire development. The quantitative method is a questionnaire based on the Kano model classifying user requirements as *must-be*, *one-dimensional* (attributes causing satisfaction when present and dissatisfaction when absent), *attractive*, *indifferent*, and *reverse* (attributes causing dissatisfaction when present and satisfaction when absent). The questionnaire was supplemented with comment fields. All respondents were physicians and nurses providing ALS at hospitals in the Central Denmark Region.

**Results:**

A total of 83 physicians and nurses responded to the questionnaire, 15 physicians and nurses were observed during ALS training, and 5 physicians were interviewed. On the basis of the Kano questionnaire, 53% (9/17) of requirements were classified as *indifferent*, 29% (5/17) as *attractive*, and 18% (3/17) as *one-dimensional*. The comments revealed 7 different categories of user requirements with noticeable differences between those of physicians and nurses: *technological challenges*, *keep track of time*, *documentation and history*, *disturbing element*, *improvement areas: functions*, *improvement areas: design*, and *better guidance*.

**Conclusions:**

The study provides recommendations to developers on the user requirements that need to be addressed when developing CPR support apps. Three features (*one-dimensional* attributes) must be incorporated in an in-hospital CPR support app: *reminder of rhythm check*, *reminder of resuscitation drugs*, and *differentiate between adults and children*. In addition, 5 features (*attractive* attributes) would result in higher user satisfaction: *all functions on one side*, *access to the patient journal in the app*, *automatic time recording when cardiac arrest is called*, *sound to guide the chest compression rate* (metronome), and *send CPR history to the DANARREST* *(Danish in-hospital cardiac arrest registry) database*.

## Introduction

New digital technologies are developing rapidly, and health care organizations are increasingly adopting and adapting to these technologies to serve clinical needs [1,2]. To mention a few benefits, such technologies support better clinical decision making, facilitate better communication, and potentially improve patient outcomes [1,3]. Among the technologies that have become commonplace within health care are mobile devices, which have led to rapid growth in the development of medical software apps [1,4-6]. These include apps used for cardiopulmonary resuscitation (CPR) guidance and support [7,8]. Most available apps used to support CPR are targeting laypersons performing basic life support [7,9].

In contrast, little is known about apps for advanced life support (ALS) during in-hospital cardiac arrests. The challenges of health care professionals with in-hospital resuscitations are dealing with high cognitive load, as they have to coordinate tasks in a team and plan timely rhythm analysis and drug administrations while considering the reversible causes of cardiac arrest [10]. Failure to adhere to guidelines may adversely impact survival [11], and consequently, apps have been suggested as potential cognitive aids to improve ALS guideline adherence [12].

However, it is important to study user requirements to improve clinical usability during resuscitation [13]. Shah and Robinson [14] argue that understanding users’ needs during development determines the success or failure of technology development. Martin et al [15] support this assertion by stating that investments in research on user requirements benefit not only the developer but also the user and the entire health care sector. The proper elicitation of requirements is more likely to aid in the development of technologies that will support and be used in clinical work. Therefore, research and development of an in-hospital CPR support app based on user requirements of health care professionals is a timely and relevant subject. The development of an efficient CPR support app will contribute to the improvement of the manner in which CPR will be performed in the future. To that end, this study aims to identify what an in-hospital CPR support app should include to meet the requirements and expectations of health care professionals by evaluating the *CprPrototype* app. This translates into the following research question: What are the user requirements for an app for in-hospital CPR support?

## Methods

### The CprPrototype App

**Figure 1:**
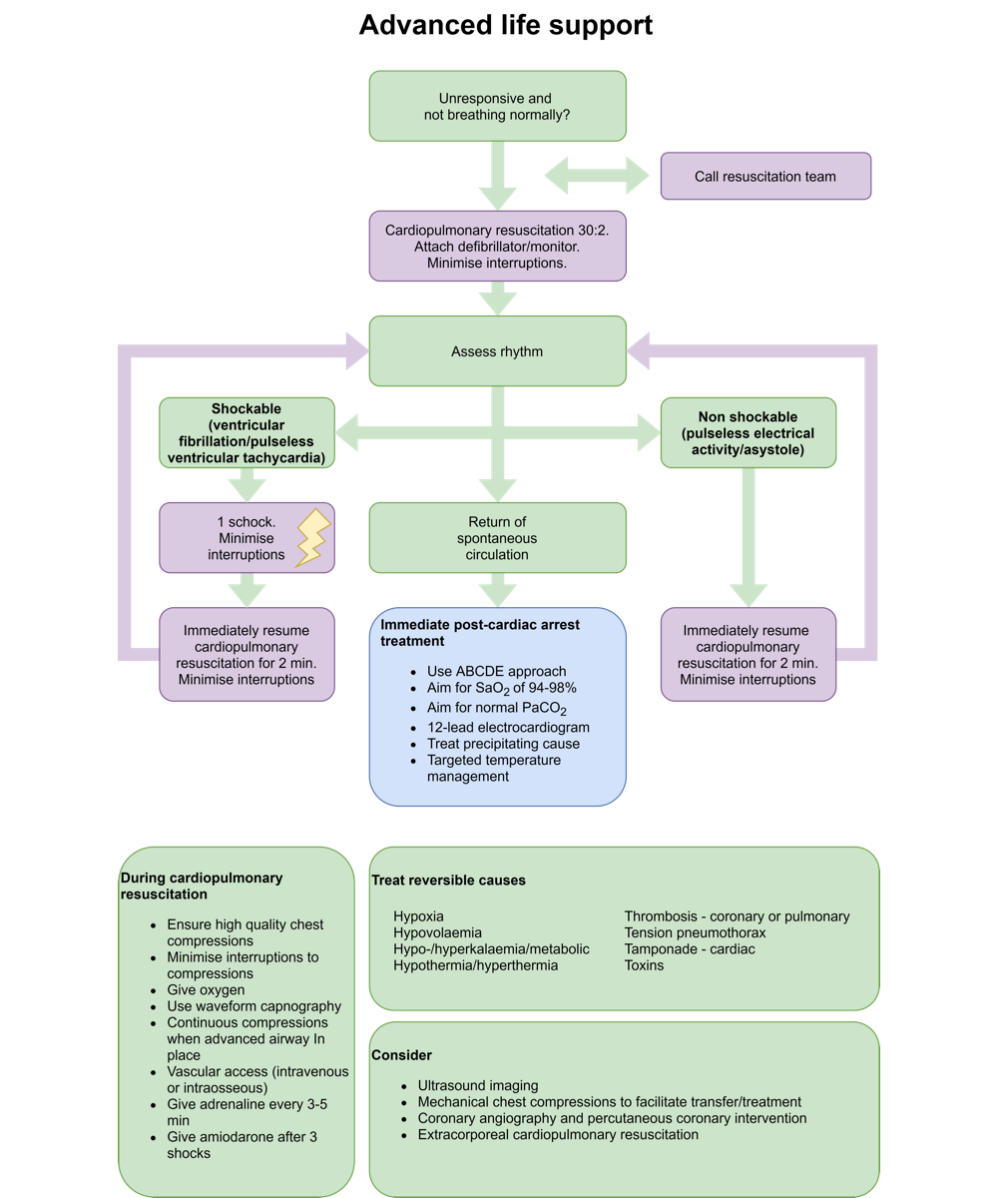
Cardiopulmonary resuscitation (CPR) algorithm.

This study seeks to elicit user requirements for an app for in-hospital CPR support by evaluating the *CprPrototype* app, developed by physicians from Aarhus University Hospital, researchers from Aarhus University, and developers from Aarhus Business Academy. The app is based on the European Resuscitation Council guideline for ALS (adapted from Soar et al [10]; Figure 1).

**Figure 2:**
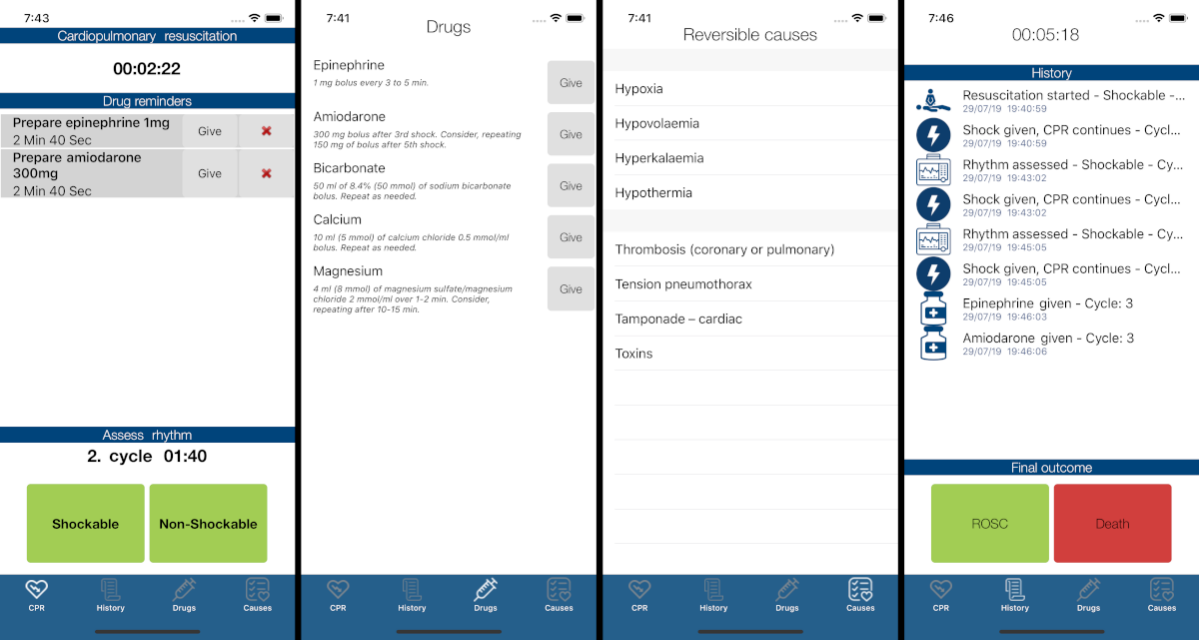
Screenshot of CprPrototype app. ROSC: Return of Spontaneous Circulation.

When performing CPR, the user of the CprPrototype app can choose the algorithm for shockable rhythms or nonshockable rhythms (screenshot 1 in Figure 2). The app then starts a 2-min cycle with a countdown for the next rhythm check. Depending on the rhythm, the app instructs the user when to prepare specific resuscitation drugs, including the dose (screenshot 2 in Figure 2). An available feature is the ability to see a list of possible reversible causes of cardiac arrest (screenshot 3 in Figure 2). The app continuously keeps track of time, and every action performed by the user in the app is tracked and stored in the app’s log (*History*) feature (screenshot 4 in Figure 2).

### Design

As the app serves to support in-hospital CPR, it is important from a development perspective to understand the needs of nurses and physicians during resuscitation. Therefore, we applied a mixed methods research design to study user requirements.

First, we observed an ALS course to understand the unspoken needs of nurses and physicians in providing ALS. The ALS course included simulations of cardiac arrests, and the participants practiced each step of ALS. During the simulations, the nurses and physicians were divided into teams. We followed each team through participant observation in an effort to uncover the needs that were not verbally articulated by the course participants. We took extensive field notes that were subsequently coded and analyzed to identify common themes [16]. The themes (resuscitation challenges, smooth collaboration, information overload, efficient communication, and need for structure and overview) provided insight into the work processes and communication of resuscitation team members, their unspoken needs for support and guidance, and helped prepare the subsequent interviews.

Second, we conducted interviews with physicians who served as resuscitation team leaders. The interviews were semistructured, based on insights from our observations. An interview guide was used to ensure the structure and comparable answers. The interviews were transcribed and analyzed through systematic text condensation to uncover their meaning [17]. The analysis facilitated our understanding of how they practice ALS, medical terminology and technology used during resuscitation, and use case scenarios for the app.

Third, we used the interview results together with the observations to develop questions for the Kano-type questionnaire. The questionnaire was sent to the respondents with an attached video of how the app functions. Furthermore, the questionnaire data were collected and managed using the REDCap (REDCap consortium; research electronic data capture) software platform [18,19].

The study complies with the ethical principles for medical research involving human subjects. According to the Danish National Committee on Biomedical Research Ethics, the study does not require approval from an ethical review committee. The study took place from February to May 2019.

### The Kano Model

To elicit user requirements and improve user satisfaction in developing health care products and services, the Kano model has previously been used [13,20,21]. The model provides insight into user requirements and whether different features (quality attributes) of a service or product contribute to greater or lower customer satisfaction [21]. Thus, it guides in prioritizing between user requirements and identifying opportunities when designing or improving products and services based on customer needs [13].

**Figure 3:**
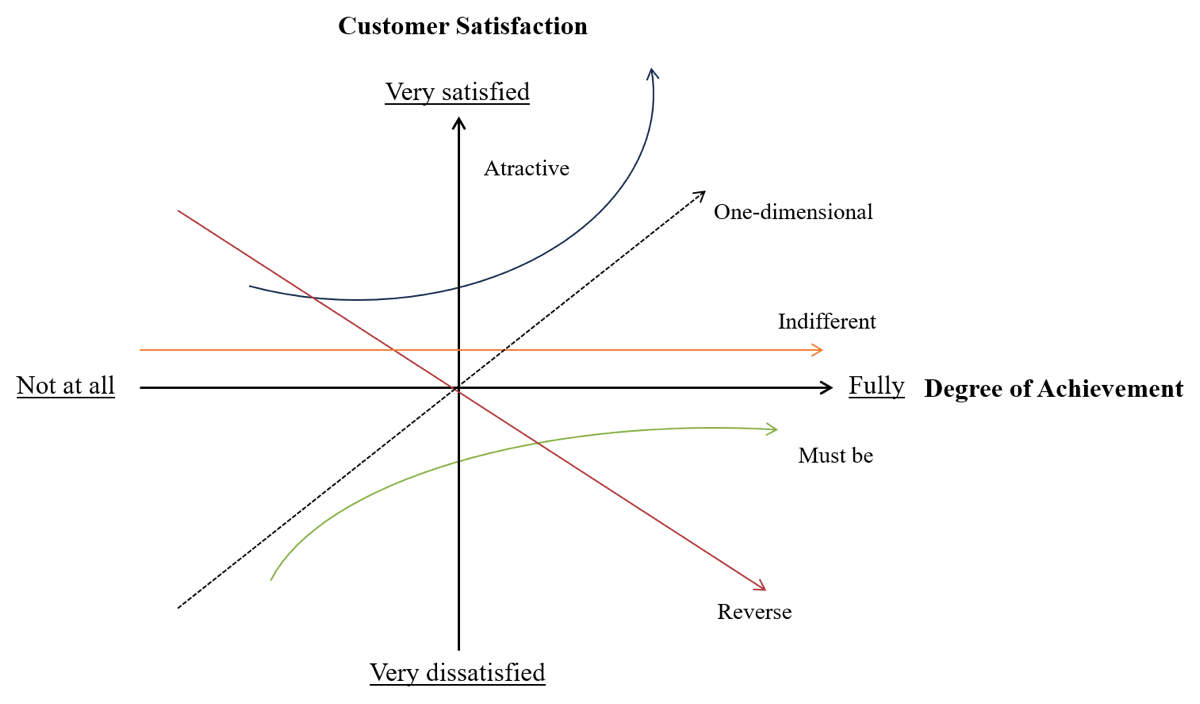
The Kano model. The horizontal axis shows the level of fulfillment of a requirement, and the vertical axis displays the level satisfaction with a requirement.

Kano et al [22] proposed a 2-dimensional quality model to classify and categorize an element of a service or product. The model is based on the motivator-hygiene theory by Herzberg et al [23], positing that the factors causing satisfaction are different from those causing dissatisfaction. The model helps visualize the relationship between the product’s functionality and customer satisfaction (Figure 3—adapted from Witell and Löfgren [24]). The model serves to explain the role of various quality attributes in determining customer satisfaction as a basis for developing a product or service.

As illustrated, the model classifies the quality attributes into 5 categories: *must-be*, *one-dimensional*, *attractive*, *indifferent*, and *reverse quality* (listed based on importance). The *must-be* attributes are expected by the customer and do not result in increased customer satisfaction, but if these attributes are not present, customers are dissatisfied. The *one-dimensional* attributes cause satisfaction when present and cause dissatisfaction when absent. The *attractive* attributes are unexpected and delight the customer, which increases customer satisfaction, but they do not cause dissatisfaction when absent, because they are not expected. Therefore, these attributes often reflect unspoken needs. Finally, the *indifferent* attributes neither cause satisfaction nor dissatisfaction, and *reverse* attributes result in dissatisfaction when present and cause satisfaction when absent [24].

We used the Kano 5-level questionnaire to classify the features into 5 categories. The features are first evaluated according to a functional question (how respondents feel if a particular feature is present) and subsequently a dysfunctional question (how respondents feel if the feature is not present). The respondents had to choose between 5 possible responses (Textbox 1).

**Example questions and possible responses from the Kano questionnaire.**

- If the app is able to differentiate between adults and children, how would you feel?
  - I like it that way
  - It must be that way
  - I am neutral
  - I can accept it to be that way
  - I dislike it that way

- If the app is not able to differentiate between adults and children, how would you feel?
  - I like it that way
  - It must be that way
  - I am neutral
  - I can accept it to be that way
  - I dislike it that way

When the survey was completed, all answers were evaluated and placed in an evaluation table [21] (Table 1) and categorized into M (must-be), O (one-dimensional), A (attractive), I (indifferent), R (reverse), and Q (questionable) quality attributes. If an answer is categorized as questionable, it indicates a conflicting answer because the respondent has answered *like* to both the functional and dysfunctional question, making the response invalid [21]. All questions from the survey are listed in the evaluation table, where an attribute is assigned according to the most frequently used response category (Tables 1, 2, and 3).

The coefficient of customer satisfaction shows how strongly a feature influences satisfaction or dissatisfaction, which helps developers prioritize user requirements. The coefficient consists of positive (satisfaction) and negative (dissatisfaction) values, and the coefficient shows the satisfaction or dissatisfaction with the presence or absence of a feature. The positive value in the formula below shows the satisfaction when a requirement is met, and the negative value shows dissatisfaction when a requirement is not met. The coefficient is calculated as follows (adapted from the study by Berger et al [25]):


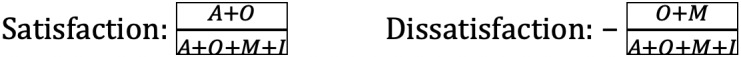


A, O, M, and I indicate the frequency of each category shown in the evaluation table. The negative sign in front of the dissatisfaction formula emphasizes the negative influence on customer satisfaction when the requirement is not met or if the feature is not part of the product [20]. Features of the evaluated product or service that yield high positive and negative values should be prioritized and addressed [21].

**Table 1:** Kano evaluation table.

Characteristics		Dysfunctional				
		Like it	Must-be	Neutral	Accept it	Dislike
**Functional**						
	Like it	Q^a^	A^b^	A	A	O^c^
	Must-be	R^d^	I^e^	I	I	M^f^
	Neutral	R	I	I	I	M
	Accept it	R	I	I	I	M
	Dislike	R	R	R	R	Q

^a^Q: questionable.

^b^A: attractive.

^c^O: one-dimensional.

^d^R: reverse.

^e^I: indifferent.

^f^M: must-be.

**Table 2:** Kano evaluation table—all results (n=83).

Questions	A^a^, n (%)	M^b^, n (%)	O^c^, n (%)	R^d^, n (%)	Q^e^, n (%)	I^f^, n (%)	Category
1. Have a phone in your hand during resuscitation	24 (29)	4 (5)	1 (1)	7 (8)	1 (1)	46 (55)	I
2. Use the app during resuscitation	33 (40)	N/A^g^	6 (7)	5 (6)	1 (1)	38 (46)	I
3. All functions on one side	28 (34)	5 (6)	19 (23)	3 (4)	1 (1)	27 (33)	A
4. Bigger text in the app	13 (16)	5 (6)	9 (11)	5 (6)	1 (1)	50 (60)	I
5. Bigger icons in the app	10 (12)	5 (6)	7 (8)	7 (8)	1 (1)	53 (64)	I
6. Color on the alarm	12 (14)	1 (1)	4 (5)	7 (8)	1 (1)	58 (70)	I
7. Reminder of rhythm check	17 (20)	16 (19)	33 (40)	4 (5)	1 (1)	12 (14)	O
8. Reminder of resuscitation drugs	15 (18)	13 (16)	35 (42)	2 (2)	2 (2)	16 (19)	O
9. Differentiate between adults and children	9 (11)	14 (17)	43 (52)	2 (2)	1 (1)	14 (17)	O
10. Sound on the alarm by the end of a 2-minute cycle	12 (14)	7 (8)	25 (30)	10 (12)	2 (2)	27 (33)	I
11. Turn of the alarm by the end of a 2-minute cycle	16 (19)	13 (16)	18 (22)	4 (5)	1 (1)	31 (37)	I
12. Vibration instead of alarm by the end of a 2-minute cycle	12 (14)	4 (5)	12 (14)	18 (22)	4 (5)	33 (40)	I
13. Access to the patient journal in the app	26 (31)	1 (1)	7 (8)	22 (27)	3 (4)	24 (29)	A
14. Automatic time recording when cardiac arrest is called	39 (47)	5 (6)	18 (22)	2 (2)	1 (1)	18 (22)	A
15. More information about the reversible causes for cardiac arrest	23 (28)	7 (8)	20 (24)	4 (5)	1 (1)	28 (34)	I
16. Sound to guide the chest compression rate (metronome)	29 (35)	4 (5)	8 (10)	19 (23)	1 (1)	22 (27)	A
17. Send CPR^h^ history to the DANARREST^i^ database	36 (43)	3 (4)	22 (27)	1 (1)	1 (1)	20 (24)	A

^a^A: attractive.

^b^M: must-be.

^c^O: one-dimensional.

^d^R: reverse.

^e^Q: questionable.

^f^I: indifferent.

^g^N/A: not applicable.

^h^CPR: cardiopulmonary resuscitation.

^i^DANARREST: Danish in-hospital cardiac arrest registry.

**Table 3:** Kano evaluation table—physicians (n=53).

Questions	A^a^, n (%)	M^b^, n (%)	O^c^, n (%)	R^d^, n (%)	Q^e^, n (%)	I^f^, n (%)	Category
1. Have a phone in your hand during resuscitation	13 (25)	1 (2)	N/A^g^	6 (11)	1 (2)	32 (60)	I
2. Use the app during resuscitation	17 (32)	N/A	5 (9)	5 (9)	1 (2)	25 (47)	I
3. All functions on one side	20 (38)	3 (6)	14 (26)	2 (4)	1 (2)	13 (25)	A
4. Bigger text in the app	7 (13)	2 (4)	6 (11)	4 (8)	1 (2)	33 (62)	I
5. Bigger icons in the app	7 (13)	3 (6)	4 (8)	6 (11)	1 (2)	32 (60)	I
6. Color on the alarm	7 (13)	N/A	2 (4)	3 (6)	1 (2)	40 (75)	I
7. Reminder of rhythm check	10 (19)	10 (19)	21 (40)	3 (6)	1 (2)	8 (15)	O
8. Reminder of resuscitation drugs	9 (17)	8 (15)	22 (42)	1 (2)	2 (4)	11 (21)	O
9. Differentiate between adults and children	5 (9)	7 (13)	28 (53)	1 (2)	1 (2)	11 (21)	O
10. Sound on the alarm by the end of a 2-minute cycle	8 (15)	4 (8)	14 (26)	6 (11)	2 (4)	19 (36)	I
11. Turn of the alarm by the end of a 2-minute cycle	11 (21)	12 (23)	11 (21)	2 (4)	1 (2)	16 (30)	I
12. Vibration instead of alarm by the end of a 2-minute cycle	8 (15)	4 (8)	7 (13)	14 (26)	3 (6)	17 (32)	I
13. Access to the patient journal in the app	19 (36)	N/A	5 (9)	17 (32)	2 (4)	10 (19)	A
14. Automatic time recording when cardiac arrest is called	26 (49)	2 (4)	11 (21)	2 (4)	1 (2)	11 (21)	A
15. More information about the reversible causes for cardiac arrest	15 (28)	4 (8)	13 (25)	2 (4)	1 (2)	18 (34)	I
16. Sound to guide the chest compression rate (metronome)	20 (38)	1 (2)	3 (6)	14 (26)	1 (2)	14 (26)	A
17. Send CPR^h^ history to the DANARREST^i^ database	27 (51)	1 (2)	12 (23)	1 (2)	1 (2)	11 (21)	A

^a^A: attractive.

^b^M: must-be.

^c^O: one-dimensional.

^d^R: reverse.

^e^Q: questionable.

^f^I: indifferent.

^g^N/A: not applicable.

^h^CPR: cardiopulmonary resuscitation.

^i^DANARREST: Danish in-hospital cardiac arrest registry.

### Questionnaire Design

**Figure 4:**
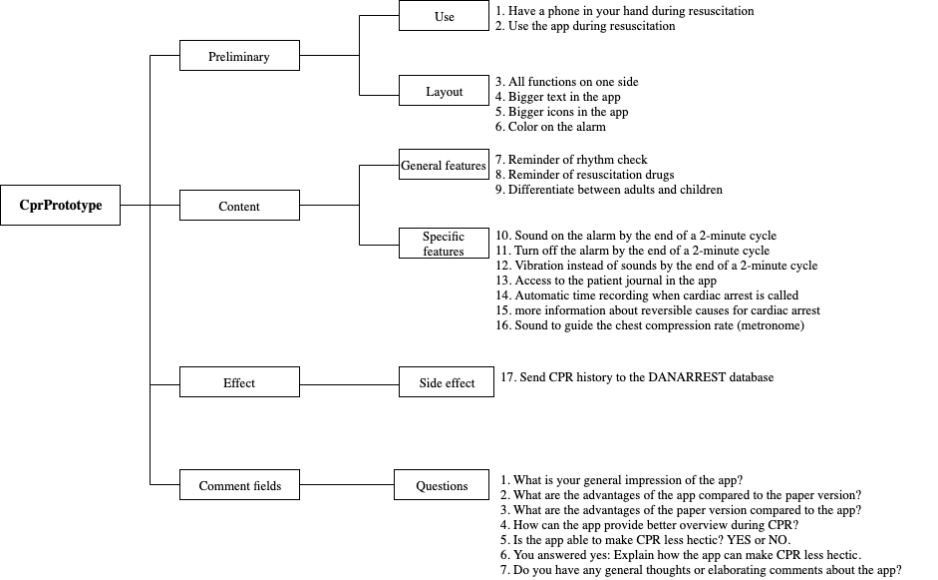
The questionnaire framework. CPR: cardiopulmonary resuscitation; DANARREST: Danish in-hospital cardiac arrest registry.

Before distributing the Kano questionnaire to respondents, a pilot test was performed with 5 physicians to evaluate their understanding of the questions. Their feedback helped us modify questions to facilitate understanding and ensure reliability and validity by adapting the wording to the terminology used by both nurses and doctors. The final questionnaire design consists of 3 main categories of questions and 5 subcategories (Figure 4).

The Kano model is used to elicit user requirements and provides insight into the needs and priorities of users, but it does not provide a more detailed explanation of those requirements. To this end, supplementary methods are needed. Therefore, we added additional questions with comment fields that allow respondents to elaborate on answers. As the study participants had not used the CprPrototype app beforehand, we created a video in Danish that explains the app and its purpose [26].

An analysis of the comments was performed to identify themes and interpret the answers to the functional and dysfunctional questions.

### Study Participants

Physicians and nurses are considered the users in this study, as they respond to in-hospital resuscitations [27].

We observed 15 physicians and nurses at an ALS course at a university hospital. In the semistructured interviews, a total of 5 physicians from different hospitals in the Central Denmark Region were interviewed. All 5 interviewees acted as team leaders during in-hospital resuscitation and provided feedback on the CprPrototype app. Finally, the questionnaire was sent to a total of 469 physicians and nurses from different hospitals in the Central Denmark Region who had previously participated in an ALS course. Questionnaires were distributed by course coordinators via email to safeguard their anonymity and ensure compliance with the General Data Protection Regulation (GDPR) [28]. The exact number of nurses compared with the number of physicians is unknown. Respondents were given the option of participating in a draw to win movie tickets.

## Results

In total, 17.7% (83/469) of physicians and nurses responded. Overall, 64% (53/83) of respondents were physicians and 36% (30/83) were nurses.

A total of 17 app features were rated and classified using the Kano evaluation table. The Kano evaluation table shows the classification of each question from the questionnaire based on user requirements (Tables 2, 3, and 4). The tables below show the difference between the individual results obtained from physicians and nurses and the aggregated results. The overall Kano analysis (Table 2) indicates that more than half of the features are classified as *indifferent* (9/17, 53%), with only 5 being *attractive* (5/17, 29%) and 3 being *one-dimensional* (3/17, 18%). None of the features are classified as *must-be*. The frequent *indifferent* classification suggests that neither the presence nor absence of most of the evaluated features makes a difference to the user. *One-dimensional* means that the absence of the feature will cause dissatisfaction. The features classified as *one-dimensional* are questions 7, 8, and 9 (Table 2). Questions 3, 13, 14, 16, and 17 (Table 2) point to *attractive* features that delight the users because they are not expected but their absence does not cause dissatisfaction.

Both physicians and nurses were *indifferent* to most of the evaluated features (9/17, 53%). There is, however, a difference in user requirements between physicians and nurses regarding the *one-dimensional* and *attractive* categories. Overall, 29% (5/17) of the features were classified as *one-dimensional* by the nurses compared with 18% (3/17) by physicians. This difference indicates that nurses to a higher degree believe that one particular feature, *sending CPR history to the DANARREST database* (the national in-hospital cardiac arrest quality registry in Denmark), needs to be incorporated in the app and will cause dissatisfaction if it is not. In turn, based on the answers by the physicians, this feature is classified as *attractive*.

**Table 4:** Kano evaluation table—nurses (n=30).

Questions	A^a^, n (%)	M^b^, n (%)	O^c^, n (%)	R^d^, n (%)	Q^e^, n (%)	I^f^, n (%)	Category
1. Have a phone in your hand during resuscitation	11 (37)	3 (10)	1 (3)	1 (3)	N/A^g^	14 (47)	I
2. Use the app during resuscitation	16 (53)	N/A	1 (3)	N/A	N/A	13 (43)	A
3. All functions on one side	8 (27)	2 (7)	5 (17)	1 (3)	N/A	14 (47)	I
4. Bigger text in the app	6 (20)	3 (10)	3 (10)	1 (3)	N/A	17 (57)	I
5. Bigger icons in the app	3 (10)	2 (7)	3 (10)	1 (3)	N/A	21 (70)	I
6. Color on the alarm	5 (17)	1 (3)	2 (7)	4 (13)	N/A	18 (60)	I
7. Reminder of rhythm check	7 (23)	6 (20)	12 (40)	1 (3)	N/A	4 (13)	O
8. Reminder of resuscitation drugs	6 (20)	5 (17)	13 (43)	1 (3)	N/A	5 (17)	O
9. Differentiate between adults and children	4 (13)	7 (23)	15 (50)	1 (3)	N/A	3 (10)	O
10. Sound on the alarm by the end of a 2-minute cycle	4 (13)	3 (10)	11 (37)	4 (13)	N/A	8 (27)	O
11. Turn of the alarm by the end of a 2-minute cycle	5 (17)	1 (3)	7 (23)	2 (7)	N/A	15 (50)	I
12. Vibration instead of alarm by the end of a 2-minute cycle	4 (13)	N/A	5 (17)	4 (13)	1 (3)	16 (53)	I
13. Access to the patient journal in the app	7 (23)	1 (3)	2 (7)	5 (17)	1 (3)	14 (47)	I
14. Automatic time recording when cardiac arrest is called	13 (43)	3 (10)	7 (23)	N/A	N/A	7 (23)	A
15. More information about the reversible causes for cardiac arrest	8 (27)	3 (10)	7 (23)	2 (7)	N/A	10 (33)	I
16. Sound to guide the chest compression rate (metronome)	9 (30)	3 (10)	5 (17)	5 (17)	N/A	8 (27)	A
17. Send CPR^h^ history to the DANARREST^i^ database	9 (30)	2 (7)	10 (33)	N/A	N/A	9 (30)	O

^a^A: attractive.

^b^M: must-be.

^c^O: one-dimensional.

^d^R: reverse.

^e^Q: questionable.

^f^I: indifferent.

^g^N/A: not applicable.

^h^CPR: cardiopulmonary resuscitation.

^i^DANARREST: Danish in-hospital cardiac arrest registry.

The comments of the respondents provide additional insight into the questionnaire responses. A total of 68 respondents answered questions with comments. Of the 68 respondents, 25 were nurses and 43 were physicians. From these comments, we identified 7 main themes in an attempt to better understand the differences in user requirements between physicians and nurses (Table 5). The column *frequency* shows how many times both physicians and nurses made a comment, mentioning one of the themes. In the columns *Physicians* and *Nurses*, we show the frequency of themes among physicians and nurses, respectively.

We quantified the comments to use the resulting values as a means to identify patterns and thereby convert them into central themes. This allows us to discern additional differences between physicians and nurses regarding user requirements.

**Table 5:** Themes of comment fields.

Theme		Frequency	Physicians (n=43), n (%)	Nurses (n=25), n (%)
Technological challenges		24	23 (53)	1 (4)
Keep track of time		33	24 (56)	9 (36)
Documentation and history		24	14 (33)	10 (40)
Disturbing element		14	12 (28)	2 (8)
**Areas of improvement**				
	Functions	19	15 (35)	4 (16)
	Design	26	15 (35)	11 (44)
Better guidance		39	22 (51)	17 (68)

The themes in Table 5 are the most dominant among all the comments, revealing that the same themes are salient across the comments of both physicians and nurses, despite differences in frequency.

A clear difference in requirements is the preference of nurses for more information and guidance in the app compared with the physicians. As shown in Table 5, 68% (17/25) of nurses preferred better guidance during CPR in comparison with 51% (22/43) of physicians. One of the main concerns expressed by physicians is technological challenges, such as the risk of the smartphone running out of battery or crashing during CPR. In total, 53% (23/43) of physicians expressed this concern, whereas only 4% (1/25) of nurses commented on this concern. The physicians also fear that the app might become a disturbance during CPR (12/43, 28%). In contrast, only a few of the nurses mentioned the same theme (2/25, 8%).

Table 6 provides an overview of the degree of satisfaction of health care professionals when a requirement is met and dissatisfaction when a requirement is not met. Furthermore, the coefficient indicates a clear priority among the app features evaluated in the study [29]. The values of the coefficients range from 0 to 1. The closer the values are to 1, the more satisfied or dissatisfied a user is with a feature [21]. If a value is greater than 0.5, a feature is assumed to be important [29].

**Table 6:** Coefficient of user satisfaction.

Questions	Positive value	Negative value
1. Have a phone in your hand during resuscitation	0.33	−0.06
2. Use the app during resuscitation	0.50	−0.07
3. All functions on one side	0.59	−0.30
4. Bigger text in the app	0.28	−0.18
5. Bigger icons in the app	0.22	−0.16
6. Color on the alarm	0.21	−0.06
7. Reminder of rhythm check	0.64	−0.62
8. Reminder of resuscitation drugs	0.63	−0.60
9. Differentiate between adults and children	0.65	−0.71
10. Sound on the alarm by the end of a 2-minute cycle	0.52	−0.45
11. Turn of the alarm by the end of a 2-minute cycle	0.43	−0.39
12. Vibration instead of alarm by the end of a 2-minute cycle	0.39	−0.26
13. Access to the patient journal in the app	0.56	−0.13
14. Automatic time recording when cardiac arrest is called	0.71	−0.28
15. More information about the reversible causes for cardiac arrest	0.55	−0.34
16. Sound to guide the chest compression rate (metronome)	0.58	−0.19
17. Send CPR^a^ history to the DANARREST^b^ database	0.71	−0.30

^a^CPR: cardiopulmonary resuscitation.

^b^DANARREST: Danish in-hospital cardiac arrest registry.

As shown in Table 6, 3 of the features display positive values of 0.63 and 0.65, which indicate a high degree of satisfaction if the requirements are met. The same features display negative values between 0.60 and 0.71, which indicates high degrees of dissatisfaction if the requirements are not met. These features are classified as *one-dimensional* in Table 2. The coefficients, therefore, show developers how to prioritize among *one-dimensional* requirements when developing the app. The coefficients also show that questions 14 and 17, which are classified as *attractive*, also lead to high degrees of satisfaction if the corresponding features are implemented.

## Discussion

### Principal Findings

The 3 attributes classified as *one-dimensional* are app features that the user explicitly wants. The 3 features are *reminder of rhythm check*, *reminder of resuscitation drugs*, and *differentiate between adults and children*. App developers should focus on these but may also benefit from implementing the *attractive* features. According to Witell and Löfgren [24], *attractive* attributes are essential when striving for quality in products and services because of the likelihood of generating user satisfaction. This is supported by the coefficient values in Table 6, which display a user satisfaction of above 0.70 for all *attractive* features. Besides, implementing *attractive* features is a low-risk strategy, as low performance with regard to such features will not increase user dissatisfaction because they are unexpected. For example, one feature that could be added to a CPR support app to increase user satisfaction is *automatic time recording when cardiac arrest is called* (0.71). Thus, app developers may use the coefficients (Table 6) to prioritize the features to be implemented when developing an in-hospital CPR support app.

An analysis of the questionnaire comments by the respondents revealed 7 central themes. Most of the comments supported the results of the Kano questionnaire. When asked if they would use the app during resuscitation, the nurses saw it as an *attractive* feature, whereas the responses of the physicians were classified as *indifferent*. More than one theme supported these categorizations. The themes *technological challenges* and *disturbing element* were commented on frequently among physicians compared with nurses, which helped explain why the physicians were *indifferent* to *using an app during resuscitation*. In contrast, nurses frequently mentioned the value of receiving better guidance provided by the app.

Furthermore, physicians frequently made suggestions regarding functionality, whereas nurses made design suggestions. A possible explanation for the differences in user requirements between physicians and nurses is the difference in experience with ALS and the different roles they have during CPR, with physicians most frequently being team leaders. Some physicians have more experience with the ALS algorithm, which could account for their suggestions regarding functionality, whereas it is difficult for nurses to comment on functionality as they are less experienced with the algorithm. The study suggests that it might be beneficial in the future to ensure configurability of the CPR support app, depending on who the user is, given the different requirements of physicians and nurses. However, further research is necessary to investigate the differences between physicians and nurses and how to accommodate their different needs.

### Comparison With Previous Work

Several studies that have applied the Kano model in a health care context have recently been published [13,21,29-34]. None of these studies use the Kano model to elicit user requirements in the development of an in-hospital CPR support app. Using the Kano model, we are able to support the claims of both Sulisworo and Maniquiz [32] and Gustavsson et al [13] that the Kano model is a practical tool to elicit different user requirements in a health care context and help prioritize between them. However, we recommend combining the Kano model with qualitative methods. We used observations and interviews to develop the Kano questionnaire, and we supplemented the Kano questionnaire with more open questions and comment fields, encouraging respondents to elaborate on their answers. This is a contribution to the existing Kano model methodology that allowed us to gain an in-depth understanding of user requirements and their priorities based on different roles (ie, physicians and nurses). The study by Gustavsson et al [13] shows the importance of incorporating the perspectives of individuals in multiple roles, which should be taken into consideration when using the Kano model. In doing so, practitioners and researchers can capture a wide range of different user needs. Our study corroborates the findings of Gustavsson et al [13] in the sense that our results show different user requirements based on the different roles of nurses and physicians.

Kalz et al [6] reported about an evaluation study of usability and quality criteria in developing an app for basic life support. In comparison, our study elicits user requirements for an ALS support app. Not only does our study offer recommendations for app developers to create value through implementation of specific *attractive* and *one-dimensional* features, but it also shows the importance of eliciting user requirements. Our results compare with those of Liao et al [34], who focus on exercise apps. Their study also recommends that app developers focus on value creation and invoke positive emotions through *attractive* features. Although there are similarities between the studies, our study extends the findings of Liao et al [34] by recommending that developers also focus on *one-dimensional* features and not only *attractive* features. They focus on *attractive* features because it enhances the strategic advantage in a competitive market with thousands of other apps similar to the one they are developing.

One study suggests that CPR support apps help improve the performance of ALS [12], although the literature on the subject is scarce. Low et al [12] found that test groups using an app during a simulation test improved the quality of ALS compared with the control group not using the app. Our study is the first to focus on app support for in-hospital CPR. Although our study elicits user requirements for such an app, we cannot provide additional support to the existing evidence that CPR support apps help improve CPR performance [12]. However, our study documents the user requirements for apps that are intended to precisely accomplish that goal.

### Limitations

A limitation of this study is the low response rate. In total, 469 potential respondents were contacted of which 83 responded, which translates into a response rate of 18%. Compliance with the European GDPR regulation necessitated that the questionnaires were distributed by health care coordinators who were in possession of the names and email addresses of the respondents. However, indirect contact with respondents limited our ability to encourage participation through personal contact and reminders. Furthermore, the study only included physicians and nurses from the Central Denmark Region. However, the Danish health care system is homogeneous. Therefore, the results are, in all probability, comparable across Danish regions.

Furthermore, this study shows challenges in eliciting user requirements for a CPR support app, as most of the features are classified as *indifferent*. One reason for the classification can be attributed to the respondents not having used the CprPrototype app in real life but only having seen it presented in a video. Consequently, they may have difficulty articulating their requirements. In the comment fields, most respondents mentioned that they would like to try the app. If the respondents try the app in a simulation, they may have more specific comments regarding the features they need. Thus, a limitation is that this study does not include a simulated or a clinical resuscitation attempt. Future research should address this limitation. Thus, clinical investigations need to be done once the user requirements have been incorporated.

### Conclusions

When developing a product or service such as an app for clinical use, focusing on user requirements is essential. Therefore, we address a knowledge gap by using the Kano model to elicit the user requirements for an in-hospital CPR support app. In total, 3 requirements classified as *one-dimensional* should be prioritized and incorporated in the app: *reminder of rhythm check*, *reminder of resuscitation drugs*, and *differentiate between adults and children*. This study also revealed 5 *attractive* requirements that should be prioritized in developing CPR apps to increase user satisfaction: *all functions on one side*, *access to the patient journal in the app*, *automatic time recording when cardiac arrest is called*, *sound to guide the chest compression rate* *(metronome),* and *send CPR history to the DANARREST database*.

Although this study shows an increasing use of mobile apps during CPR and highlights the importance of eliciting user requirements, our study is uniquely able to provide recommendations to developers on the specific user requirements that should be addressed when developing CPR support apps.

Looking toward the future, it will be important to ensure that the CprPrototype app complies with the European Union Medical Device Regulation of 2017 [35] and relevant national legislation before it can be used in clinical medical practice without fear of personal liability [36]. The next step is to integrate the app with widely used defibrillators into a coherent CPR decision support system for monitoring physiological processes and guiding CPR based on dynamic algorithms. This integration will pave the way for systems interoperability, so the information can be used in, for example, digital hospital command centers [37].

## Abbreviations

ALS: advanced life support
CPR: cardiopulmonary resuscitation
DANARREST: Danish in-hospital cardiac arrest registry
GDPR: General Data Protection Regulation
